# Characterization of the Antitumor Potential of Extracts of *Cannabis sativa* Strains with High CBD Content in Human Neuroblastoma

**DOI:** 10.3390/ijms24043837

**Published:** 2023-02-14

**Authors:** Laura Sánchez-Sánchez, Javier García, Roberto Fernández, Ekaterina Noskova, June Egiguren-Ortiz, Marina Gulak, Eneko Ochoa, Antonio Laso, Mikel Oiarbide, José Ignacio Santos, María Fe Andrés, Azucena González-Coloma, Albert Adell, Egoitz Astigarraga, Gabriel Barreda-Gómez

**Affiliations:** 1 Research and Development Department, IMG Pharma Biotech S.L., 48160 Derio, Spain; 2 Instituto de Biología y Genética Molecular (IBGM), Unidad de Excelencia, Universidad de Valladolid-CSIC, 47011 Valladolid, Spain; 3 Institute of Agricultural Sciences (ICA), Spanish Research Council (CSIC), 28006 Madrid, Spain; 4 Instituto de Biomedicina y Biotecnología de Cantabria (IBBTEC), Consejo Superior de Investigaciones Científicas (CSIC), University of Cantabria, 39011 Santander, Spain; 5 Pharmacology Department, Faculty of Medicine and Nursing, University of the Basque Country UPV/EHU, 48940 Leioa, Spain; 6 Cruz Roja Hospital, 48001 Bilbao, Spain; 7 Research and Development Division, AleoVitro, 48160 Derio, Spain

**Keywords:** cannabis, extracts, neuroblastoma, antitumor

## Abstract

Cannabis has been used for decades as a palliative therapy in the treatment of cancer. This is because of its beneficial effects on the pain and nausea that patients can experience as a result of chemo/radiotherapy. Tetrahydrocannabinol and cannabidiol are the main compounds present in *Cannabis sativa*, and both exert their actions through a receptor-mediated mechanism and through a non-receptor-mediated mechanism, which modulates the formation of reactive oxygen species. These oxidative stress conditions might trigger lipidic changes, which would compromise cell membrane stability and viability. In this sense, numerous pieces of evidence describe a potential antitumor effect of cannabinoid compounds in different types of cancer, although controversial results limit their implementation. In order to further investigate the possible mechanism involved in the antitumoral effects of cannabinoids, three extracts isolated from *Cannabis sativa* strains with high cannabidiol content were analyzed. Cell mortality, cytochrome c oxidase activity and the lipid composition of SH-SY5Y cells were determined in the absence and presence of specific cannabinoid ligands, with and without antioxidant pre-treatment. The cell mortality induced by the extracts in this study appeared to be related to the inhibition of the cytochrome c oxidase activity and to the THC concentration. This effect on cell viability was similar to that observed with the cannabinoid agonist WIN55,212-2. The effect was partially blocked by the selective CB1 antagonist AM281, and the antioxidant α-tocopherol. Moreover, certain membrane lipids were affected by the extracts, which demonstrated the importance of oxidative stress in the potential antitumoral effects of cannabinoids.

## 1. Introduction

Cannabis belongs to a family of psychoactive plants, which is composed of different varieties, including *Cannabis sativa*, *Cannabis indica*, and *Cannabis ruderalis.* This family contains hundreds of specific compounds called cannabinoids or phytocannabinoids [1]. The most abundant components of these compounds are tetrahydrocannabinol (Δ^9^-THC) and cannabidiol (CBD) [2], which display different psychoactive capacities. These cannabinoids show antioxidant activity [3], as well as antipsychotic, neuroprotective [4], and antitumoral effects [5,6]. For example, CBD presented beneficial effects in neuronal and inflammatory diseases [5], such as Parkinson’s disease (PD), Alzheimer’s disease [7], refractory epilepsy [8], inflammatory skin diseases, and inflammatory bowel disease.

Cannabinoid compounds are lipophilic compounds, which can be ligands for the cannabinoid receptors CB1 and CB2 [1,9]. These receptors belong to the family of G-protein-coupled receptors (GPCR). The CB1 receptor is highly concentrated in brain regions [10], but it has also been identified in other tissues, such as the spleen, liver, lungs, and prostate [1]. In regard to the CB2 receptor, it has been found in immune cell types, peripheral tissues [10,11], and even in the central nervous system [1]. The activation of these receptors can be achieved by either natural or synthetic cannabinoids, from either animal or plant origin, and entails the stimulation of specific enzymatic pathways, such as protein kinase pathways, phosphoinositide-3-kinase (PI3K) pathways, or cyclooxygenase-2 (COX-2) pathways [1,12]. Synthetic cannabinoids include hundreds of compounds, such as WIN55,212-2, a full cannabinoid agonist of both CB receptors, and AM281, a specific CB1 antagonist.

Several mechanisms are involved in cannabinoids’ neuroprotective [4,13] and antioxidant effects [14]. Among the most important are their actions on mitochondrial activity. Although CB receptors are mainly located at the plasmatic membrane [15], they can also be present in mitochondrial membranes. In this sense, approximately 30% of neuronal mitochondria display CB1 in their external membranes [16]. This intracellular localization reveals the crucial role of cannabinoid receptors, in both the regulation of energy homeostasis—which is achieved by modulating the mitochondrial electron transport chain (mETC)—and in the specific modulation of physiological processes, such as learning. The activation of the mitochondrial CB1 receptor pathway involves the G Gαi protein, soluble-adenylyl cyclase (sAC), and protein kinase A (PKA) [17]. However, a non-receptor-mediated mechanism is also involved in the modulation of oxidative phosphorylation (OXPHOS) that is triggered by cannabinoids [18,19,20].

The effects of cannabinoids have been widely studied, as they can be used as possible modulators of different pathways or even of the immune response. In this sense, cannabidiol has been identified as a possible modulator of the neuroimmune axis, reducing gut inflammation in ulcerative colitis patients [21]. Moreover, as cannabinoid receptors have been identified in numerous tissues, both healthy and pathologic tissues, the ligands of CB1 and CB2 can be used to ameliorate some diseases’ symptoms or even their development. For example, both receptors have been altered in patients with Alzheimer’s disease, Parkinson’s disease, and multiple sclerosis, among others [22]. In particular, an increase in the density of both receptors has been observed in the basal ganglia of PD patients [22,23], and an increase in the CB2 receptors has also been observed in the dopaminergic neurons of the substantia nigra [22]. Furthermore, the higher expression of cannabinoid receptors has also been identified in tumors, which has been related to their malignancy [24]. As cancer is one of the main causes of mortality today, the discovery of new antitumor molecules or combinations of molecules is essential. In this sense, certain plant-derived compounds, such as curcumin [25,26], thymoquinone [25,27], isoflavones [28], resveratrol [28], or cannabinoid compounds [29], have shown interesting antitumor potential. For example, WIN55,212-2 and THC—a synthetic and natural cannabinoid, respectively—have been reported to inhibit the progression of malignant gliomas by potentiating apoptosis, which has been mediated by the CB2 receptor [24,30]. The induction of apoptosis has also been achieved by diverse cannabinoids. This effect has been observed in in vitro assays on pancreatic adenocarcinoma cell lines, glioblastoma cell lines, breast adenocarcinoma cell lines, astrocytoma cell lines, and neuroblastoma cell lines [29], all via the effect of the CB2 receptor. In addition, CBD has also been seen to induce apoptosis through an oxidative-stressed mechanism, which increases reactive oxygen species and produces the release of cytochrome c from the mitochondria [31]. Nevertheless, even though pure cannabinoids have been tested, the effect of whole cannabis extracts in cancer therapy remains elusive.

To address this question, in this study, we exposed the human neuroblastoma cell line to various *Cannabis* plant extracts, both with and without WIN55,212-2 and AM281. Moreover, the effects of pre-treatment with antioxidant compounds, both with and without extracts or synthetic cannabinoids, were tested to elucidate the relationship between the cannabinoid-induced cancer cells’ apoptosis and oxidative stress, as antioxidant therapy is often used in combination with chemotherapy or other antitumoral treatments [32,33].

## 2. Results

### 2.1. High-Pressure Liquid Chromatography of Cannabis sativa Plant Extracts

Different relative areas were achieved for cannabidiol, cannabidiolic acid (CBDA), tetrahydrocannabinol, and tetrahydrocannabinolic acid (THCA) in every *Cannabis* plant extract, as indicated in the following table (Table 1).

### 2.2. Viability of Neuroblastoma Human Cell Line against Different Treatments

Characterization of the cells’ viability was performed in order to determine the effect of the different treatments: the antioxidant compound α-tocopherol (1 μM), synthetic cannabinoids AM281 (3.5 μg/mL), and WIN55,212-2 (3.5 μg/mL), or a combination of these.

**Figure 1:**
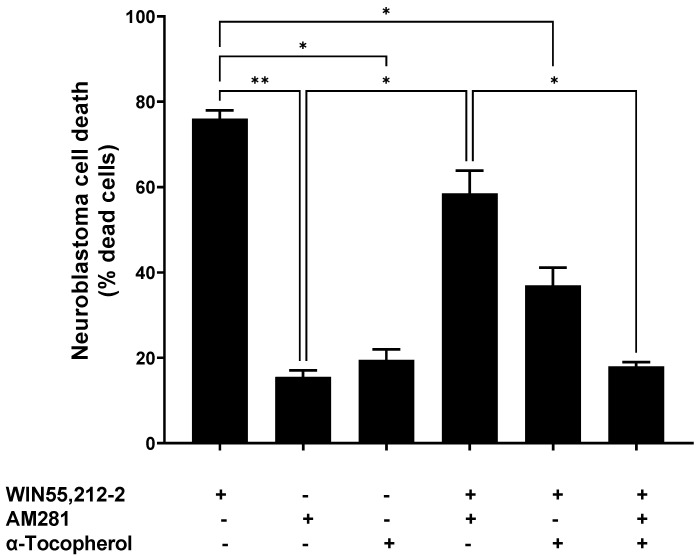
Neuroblastoma cell death upon different treatments with synthetic cannabinoids (3.5 μg/mL AM281 and 3.5 μg/mL WIN55,212-2), with or without α-tocopherol pre-treatment (1 μM). Statistical test Brown Forsythe and Welch ANOVA test was done with a Dunnett post-hoc with α set as 0.05. *p*-value < 0.05 (*); *p*-value < 0.01 (**).

The cells treated with WIN5,212-2 reached a cell death percentage of 76.0% ± 2.0, whereas AM281, an antagonist of the CB1 receptor, achieved 15.5% ± 1.6 cell death. When both of the cannabinoid molecules were incubated together, a partial reduction in cell death (58.5% ± 5.36) was observed, compared to that produced by WIN55,212-2 alone (Figure 1).

The α-tocopherol pre-treatment reduced the cell death triggered by WIN55,212-2—it dropped from 76.0% ± 2.0 to 37.0% ± 4.2. Moreover, this reduction in the WIN55,212-2-evoked cell death was higher when α-tocopherol and AM281 were used together, at 18.0% ± 1.0. This was similar to the cell death obtained when AM281 or α-tocopherol were assayed individually (15.5% ± 1.5 and 19.5% ± 2.5, respectively).

### 2.3. Viability of Neuroblastoma Human Cell Line When Mediated by Cannabinoid Extracts

Regarding the effects of the *Cannabis* extracts from different plant varieties, the cell death of the neuroblastoma cell cultures was monitored every 24 h during their treatment with the plant extracts. This was both with the presence and absence of 3.5 μg/mL AM281 and 3.5 μg/mL WIN55,212-2, and both with and without pre-treatment with 1 μM α-tocopherol.

**Figure 2:**
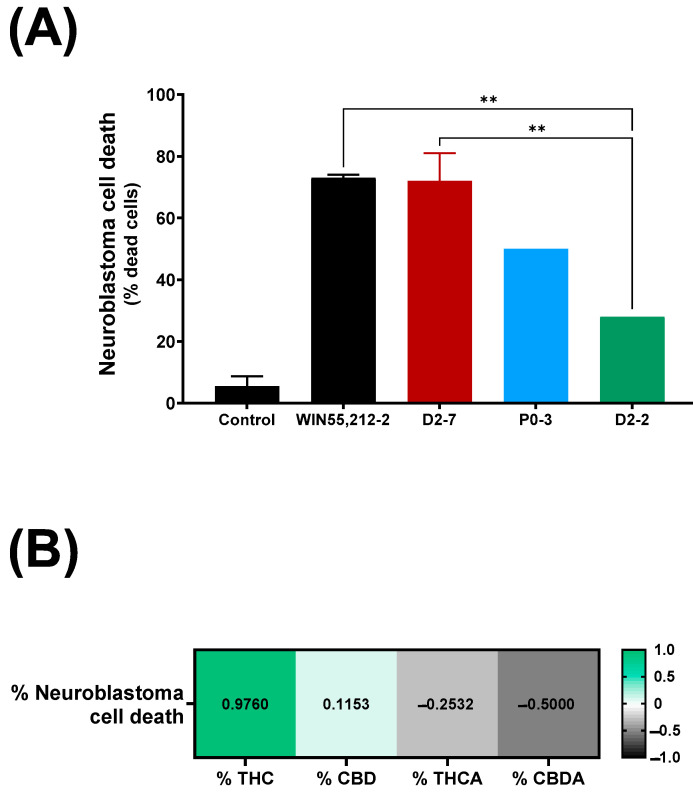
(**A**) Neuroblastoma cell death upon exposure to different *Cannabis* plant extracts (final concentration of each plant extract was 3.5 μg/mL), in control situation and exposed to WIN55,212-2 synthetic cannabinoid (3.5 μg/mL). Each condition was analyzed per duplicate. A one-way ANOVA with Tukey’s post-hoc was performed, with α set as 0.05, two-tailed. *p*-value < 0.01 (**). (**B**) Pearson correlation between neuroblastoma cell death expressed as percentage, and quantity of tetrahydrocannabinol, cannabidiol, tetrahydrocannabinol A (THCA), and cannabidiol A (CBDA), expressed as percentage.

The exposure of neuroblastoma cells to a D2-7 extract, at a concentration of 3.5 μg/mL, reached 72% ± 9 cell death (Figure 2A), whereas the same concentration of the extracts of P0-3 and D2-2 reached 50% and 28%, respectively. The differences between WIN55,212-2 and the extracts were only reached in the case of D2-2, which achieved 40% less cell death. Moreover, a positive correlation was also observed between the tetrahydrocannabinol content of an extract and neuroblastoma cell death (Figure 2B).

Significant differences were observed between the D2-7 extracts and the others in the presence of AM281. Under this condition, the D2-7 extracts achieved a cell death rate of 60% ± 1, which was 12% lower than that achieved by the control group. P0-3 showed a different effect to D2-7 but not D2-2. The P0-3 cannabinoid treatment achieved 18% ± 3.8 cell death, which was almost half the level of neuroblastoma cell death that was observed in the control (32% less cell death). Moreover, D2-2 only reached 12% ± 2 cell death, which was 16% less than the control.

**Figure 3:**
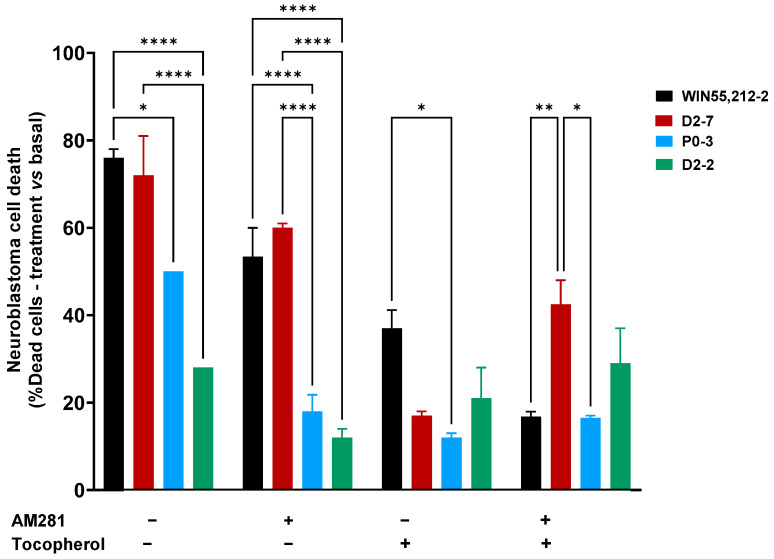
Neuroblastoma cell death at 24 h, after different treatments, both with the presence and absence of synthetic cannabinoid WIN55,212-2, and with a variety of *Cannabis* plant extracts (D2-7, P0-3, and D2-2), with or without antioxidant pre-treatment. The two-way ANOVA with Tukey’s post-hoc was performed with α set as 0.05, two-tailed. *p*-value < 0.05 (*); <0.01 (**); <0.0001 (****).

The α-tocopherol pre-treatment reduced the cell death rate in all cases, and no differences among the plant extract treatments were observed, with cell death rates of 17% ± 1, 12% ± 1, and 21% ± 7 in D2-7, P0-3, and D2-2, respectively. A reduction in cell death was observed in every plant extract, with a decrease of 76.3% in D2-7, 76% in P0-3, and 25% in D2-2. Moreover, a combination of both α-tocopherol pre-treatment and AM281 treatment, alongside the different extracts, presented differences between D2-7 and P0-3. The two cannabinoids reached 46.5% ± 5.5 and 16.5% ± 0.5, respectively, while D2-2 did not show any significant differences, reaching 29% ± 8 cell death (Figure 3). In addition, the α-tocopherol pre-treatment used alongside the AM281 treatment decreased by 17.5% in the case of D2-7 and by 17% in the case of D2-2, which was a slight increase of 1.5%.

### 2.4. Cannabis sativa Plant Extract-Mediated Cytochrome c Inhibition

**Figure 4:**
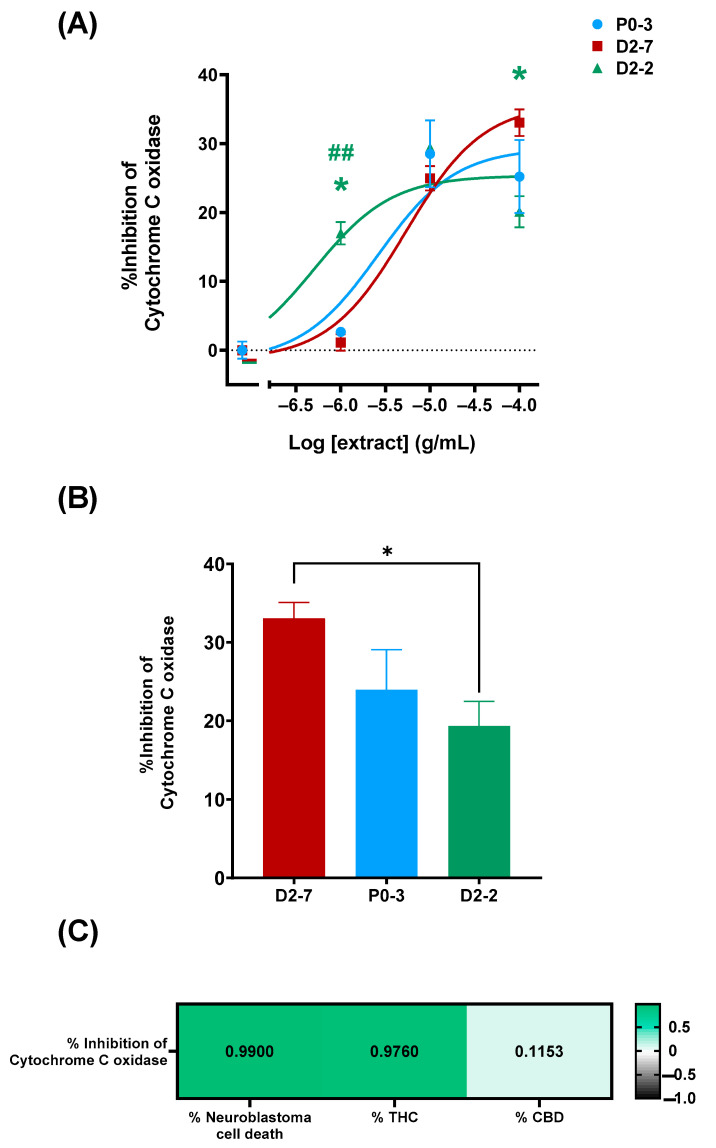
Inhibition of cytochrome c oxidase, mediated by different *Cannabis sativa* plant extracts. (**A**) Dose–response curve of P0-3, D2-7, and D2-2 plant extracts, expressed as percentage of inhibition. Non-linear regression analysis was performed using different curve fits for each dataset. As statistical analysis, two-way ANOVA statistical test and Tukey post-hoc analysis were performed on the whole dataset; α was set at 0.05, two-tailed. Analysis of P0-3 is expressed with an asterisk (*) and analysis of D2-7 is expressed with a hash (#) *p*-value < 0.05 (*); <0.01 (##). (**B**) Inhibition percentage of P0-3, D2-7, and D2-2 at the whole extract concentration of 100 μg/mL. The group compared with them is indicated by the color code. As statistical analysis, one-way ANOVA statistical test was performed, α was set at 0.05, two tailed. *p*-value < 0.05 (*). (**C**) Pearson correlation test was performed between cytochrome c oxidase inhibition percentage, cell viability (expressed as percentage of neuroblastoma cell death), tetrahydrocannabinol content (expressed as percentage of THC content), and cannabidiol content. α was set at 0.05, two-tailed, and correlation was expressed as R-Pearson value.

The characterization of the plant extracts’ effects on cytochrome c oxidase activity was performed both in the absence and the presence of plant extracts at different concentrations (1 μg/mL, 10 μg/mL, and 100 μg/mL). This was performed in order to elucidate whether or not the differences in the cell mortality rates could be due to their effects on cytochrome c oxidase (Figure 4A). The enzymatic activity was measured for 16 h, using a cell homogenate in a reaction solution and without plant extracts as a control. Cytochrome c inhibitions of 33.07% ± 2.03, 23.96% ± 5.09, and 19.35% ± 3.12 were achieved with D2-7, P0-3, and D2-2, respectively, at concentrations of 100 μg/mL (Figure 4B). Moreover, there was found to be a positive correlation between the quantity of tetrahydrocannabinol and cell death. The quantity of cannabidiol did not show a correlation with cell death.

### 2.5. Cannabis Plant Extracts’ Effects on Cell Lipidome

**Figure 5:**
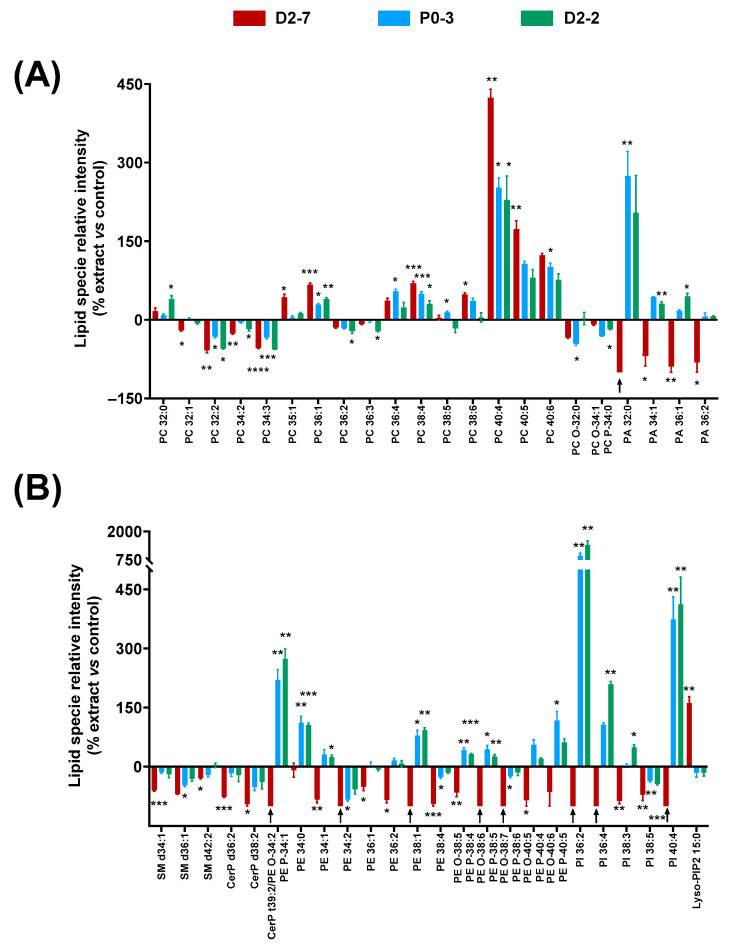
Changes in lipid fingerprint due to exposure to *Cannabis sativa* plant extracts, expressed as percentage of relative intensity of each extract with respect to the control. (**A**) Glycerophospholipids (PC, PC-O, PC-P, and PA). (**B**) Sphingolipids and glycerophospholipids (SM, CerP, PE, PE-O, PE-P, PI, and Lyso-PIP2). Normality was tested for each lipid species separately by Bartlett test; α was set at 0.05. Student t-test or Wilcoxon test were performed on the parametric or non-parametric samples, respectively. The tests were two-tailed; α was set at 0.05. *p*-value < 0.05 (*); <0.01 (**); <0.001 (***); and <0.0001 (****). Black arrows indicate the absence of a lipid species in this sample. Abbreviations: glycerophosphocholine, ether and plasmalogen forms—PC, PC-O, and PC-P); glycerophosphatidic acid—PA; sphingomyelin—SM; ceramide phosphate—CerP; glycerophosphoethanolamine, ether form and plasmalogen form—PE, PE-O, and PE-P; glycerophosphatidilinositol—PI; and lysophosphoinositol bisphosphate—Lyso-PIP2.

The effects of the plant extracts on the cell homogenates’ lipidome were studied by analyzing the lipid fingerprints of the cell homogenates that were exposed to the *Cannabis sativa* plant extracts, both with and without α-tocopherol pre-treatment. A distinguishable lipid fingerprint was observed because of their exposure to the extracts. In addition, differences were observed among the extracts (Figure 5).

Regarding the glycerophospholipids, significant differences were observed among several long-chain PCs—between 32 and 40 carbons—in the cells that were exposed to the extracts, compared to controls. PCs with more than 36 carbons generally increased: the higher the number of carbons, the higher the relative enhancement. However, this effect seemed to be reduced when the unsaturation number increased, e.g., PC 40:4 reached 423% when exposed to D2-7, whereas PC 40:5 and PC 40:6 only reached 173% and 123%, respectively. A similar effect was observed in the case of PC 38:4 and PC 38:6. By contrast, PCs with less than 36 carbons generally decreased, except in the case of PC 32:0.

In the case of the PA species, a clear difference was observed between D2-7 and the other extracts. While the D2-7 extract generally triggered a decrease, P0-3 and D2-2 induced an upregulation. This discrepancy was particularly high in PA 32:0 (Figure 5A). A similar effect was observed in the PE species and their ether versions, in which the D2-7 extract generally promoted a reduction in these lipid species, whereas, with the other plant extracts, they increased, except in the case of PE 34:2 and PE 38:4. Moreover, this discrepancy between the D2-7 extract and the others was also found in the following phosphoinositols: PI 36:2, PI 36:4, PI 38:4, and PI 40:4. The only exception here was for PI 38:5, which was reduced by all three of the extracts assayed. In contrast, lysophosphatidilinositol 15:0 was enhanced in the cells exposed to D2-7, whereas, with the other extracts, it was unchanged or even slightly decreased. This Lyso-PIP2 contains pentadecanoic fatty acid.

Regarding sphingolipids and ceramide phosphates, the three extracts appeared to promote a reduction in SM d34:1, SM d36:1, SM d42:2, CerP d36:2, and CerP d38:2. The D2-7 extract was the one that induced the greatest effect (Figure 5B).

### 2.6. Protective Effects of α-Tocopherol on Cannabis Plant Extract-Mediated Lipid Changes

**Figure 6:**
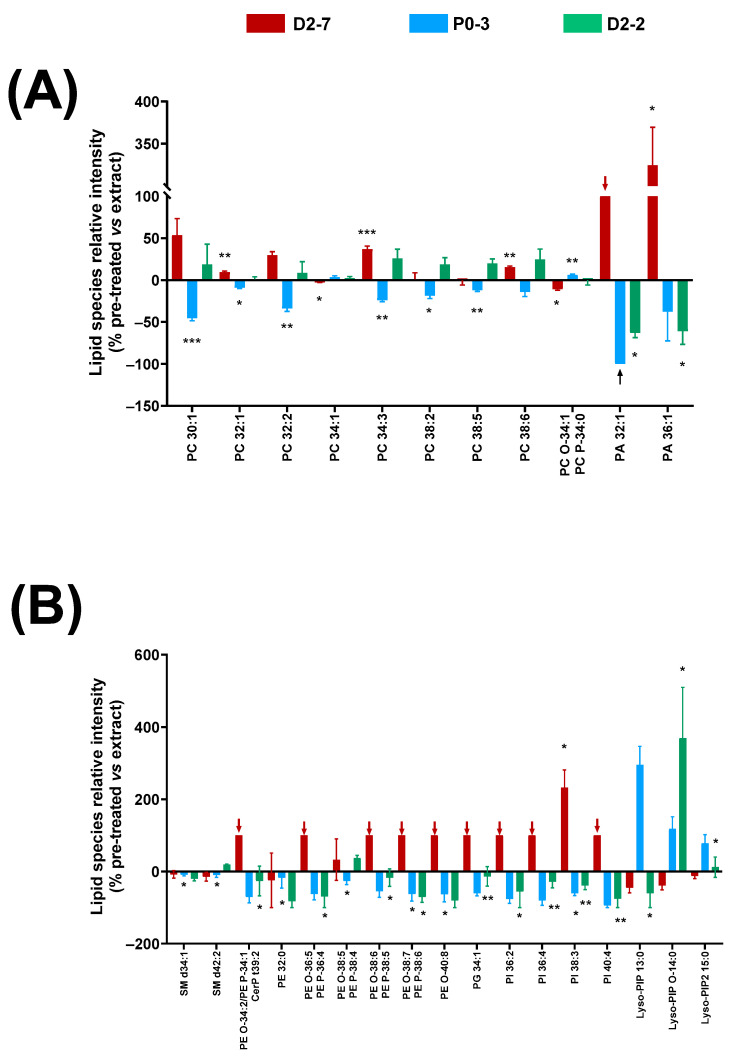
Changes in lipid fingerprint due to α-tocopherol pre-treatment in cell homogenates of cells exposed to *Cannabis sativa* plant extracts, expressed as percentage of the relative intensity of each extract, compared to the control condition. (**A**) Glycerophospholipids (PC, PC-O, PC-P, and PA). (**B**) Sphingolipids and glycerophospholipids (SM, CerP, PE, PE-O, PE-P, PG, PI, Lyso-PIP, and Lyso-PIP2). Normality was tested for each lipid species separately by Bartlett test; α was set at 0.05. Student t-test or Wilcoxon test were performed on the parametric and non-parametric samples, respectively. The tests were two-tailed and α was set at 0.05. *p*-value < 0.05 (*); <0.01 (**); and <0.001 (***). Black arrows indicate absence of a lipid species in this sample. Abbreviations: glycerophosphocholine, ether and plasmalogen forms—PC, PC-O, and PC-P; glycerophosphatidic acid—PA; sphingomyelin—SM; ceramide phosphate—CerP; glycerophosphoglycerol—PG; glycerophosphoethanolamine, ether form and plasmalogen form—PE, PE-O, and PE-P; glycerophosphatidilinositol—PI; lysophosphoinositol phosphate—Lyso-PIP; and lysophosphoinositol bisphosphate—Lyso-PIP2.

The α-tocopherol pre-treatment in the cell lines exposed to these plant extracts led to lower cell mortality rates at 24 h. Thus, this decrease in cell mortality might entail changes in the lipidic fingerprints of the cell homogenates. Different effects on the lipid relative abundance were observed for every *Cannabis* plant extract (Figure 6).

Significant changes were detected in the glycerophosphocholine species with between 30 and 38 carbons, with respect to the groups that were exposed to the antioxidant pre-treatment compared to the groups that were only treated with the extracts. This change in relative abundance was different depending on the extract used: there was a decrease in the cells exposed to the P0-3 extract, whereas an increase was observed in the D2-7 and D2-2 extracts. However, an exception was seen in PC 34:1 and its ether version, which were reversed. In regard to the PA species, the α-tocopherol pre-treatment reduced PA 36:1 and PA 32:1 in the cultures treated with the D2-2 and P0-3 extracts. In particular, PA 32:1 was absent in the pre-treated samples that were exposed to P0-3 (black arrow in Figure 6A). The opposite effect was observed in the cells that were exposed to D2-7, in which an increase in PA 32:1 and PA 36:1 was detected in the α-tocopherol pre-treated samples. Furthermore, PA 32:1 was only present in the cells that were pre-treated with α-tocopherol and exposed to D2-7, and not in those that were treated with this extract alone, i.e., without the antioxidant pre-treatment (red arrow in Figure 6A).

As in the case of the PA species, a similar effect was observed in the phosphoethanolamine species and their ethers, phosphoglycerol, and phosphoinositols with α-tocopherol. These lipid species were only present in the cells that were exposed to the D2-7 extract and pre-treated with α-tocopherol, whilst they decreased in those exposed to P0-3 and D2-2 extracts. In contrast, various lysophosphatidylinositol species displayed a general increase in the pre-treated cells that were exposed to the P0-3 extract, and a general decrease in the pre-treated cells that were exposed to D2-7. No general trend was observed in the cells that were treated with α-tocopherol and the D2-2 extract. In particular, when using this extract, a decrease was found in Lyso-PIP 13:0, an increase was found in Lyso-PIP O-14:0, and no change was found in Lyso-PIP2 15:0, compared to the group without antioxidant pre-treatment.

## 3. Discussion

In the present study, we characterized the antitumoral effects of different extracts from a variety of *Cannabis* strains, on a human model of neuroblastoma: the SH-SY5Y cell line. We also used synthetic cannabinoid ligands for reference and α-tocopherol as an antioxidant. The nonselective full cannabinoid agonist WIN55,212-2 evoked 76% cell death. However, this was slightly inhibited by the specific CB1 antagonist/inverse agonist AM281, which suggests that other mechanisms besides the CB1 receptor pathway are also involved. In this sense, α-tocopherol reduced WIN55,212-2-mediated cell death by approximately one half when it was tested alone, and WIN55,212-2-mediated cell death was reduced to the basal level when combined with AM281, which highlights the importance of oxidative stress in the antitumor effects of cannabinoids on this human neuroblastoma cell line.

Once the response of the neuroblastoma cells to the reference compounds was determined, three extracts of *Cannabis sativa* strains with high CBD content (previously characterized in a study on bovine heart membranes [34]) were studied. A variety of D2-7 extracts reached similar cell death rates as WIN55,212-2; however, P0-3 caused 28% less cell death and D2-2 caused almost 44% less cell death. Moreover, a correlation was observed between the cell mortality rate achieved and the THC content in these plant extracts; however, no correlation was observed with either CBD or the other compounds analyzed. In previous studies on breast and lung tumor cell lines, THC was seen to have a dose-dependent, inhibitory cell proliferation effect [35,36] through its actions on the EGFR pathway. However, this dose-dependent effect has not been observed in other studies on tumoral cell lines, which exposed the cells to plant extracts with high THC content; however, the higher inhibition of cell growth was described [37].

In our study, in addition to the correlation between the cell mortality rate and the THC content, we also found a positive correlation between the concentration of THC present in the three extracts of the *Cannabis sativa* strains and the inhibition of cytochrome c oxidase activity in the SH-SY5Y cell line. This correlation was also observed in a previous study on bovine heart membranes [34]. Furthermore, several studies have observed that cannabinoids modulate energy metabolism via both receptor-mediated mechanisms and non-receptor-mediated mechanisms [17,18,19,20]. Moreover, cannabinoids have also been seen to be involved in mitochondria-related toxicity [38] and oxidative stress [18]. Indeed, in other studies, the increase in the production of reactive oxygen species in both normal and tumor cells—triggered by cannabinoids [4,34]—can lead to lipid changes in cell membranes, such as lipid peroxidation.

In this context, we found that long-chain PCs that contained polyunsaturated fatty acids (PUFAs) were increased in the plant extract-treated cells, particularly PC 38:5 and PC 38:6. These data agree with the elevation of these long-chain PCs that was reported after situations of oxidative stress in [39]. By contrast, in our study, PCs with three unsaturations or less generally decreased due to the action of the *Cannabis* plant extracts. Long-chain PCs’ unsaturated fatty acids are related to the suppression of pro-inflammatory cytokines [40,41], such as TNF α [40,42], which induces the activation of apoptotic or necrotic pathways [43]. The shift in the relative abundance of the PC species that was evoked by the different cannabinoid extracts was differentially affected by the pre-treatment with α-tocopherol. The cells that were pre-treated with this antioxidant and exposed to the P0-3 extract displayed a reduction in certain PC species. No effect with respect to non-pre-treated cells was detected in the other extracts. The opposite effect was also observed between the D2-7 extract and the others with respect to the PAs, PEs, and PIs that underwent the α-tocopherol pre-treatment. This extract evoked a significant increase in Lyso-PIP2 15:0, which was not reversed by the antioxidant pre-treatment. Furthermore, certain species of lysophosphatidylinositol that contain tridecanoic, myristic, and pentadecanoic fatty acids also showed differential behavior, depending on the extract used and the α-tocopherol pre-treatment. These Lyso-PI species are the endogenous ligands of the GPR55 receptor, which can promote the activation of a PI3K-Bmx-PLCγ cascade, and a phospholipase that is involved in the dissemination of tumor cells [44,45].

Regarding sphingolipid content, the sphingomyelin and ceramide phosphate species were decreased in all of the plant extract treatments. Consistent with these results, the activation of the CB1 receptor by THC has been reported to produce the hydrolysis of cell membrane sphingomyelin, which leads to ceramide accumulation and apoptosis [46]. In other studies, an acute or sustained increase in ceramide content has been described in various cell types after cannabinoid treatment, except for neurons, which seem to be resistant to this induction [47]. However, in the activation of the apoptotic pathway, which is triggered by cannabinoid extracts, the reduction in ceramide phosphates may also be important, as these ceramide metabolites exhibit substantial mitogenic and anti-apoptotic properties [48].

## 4. Materials and Methods

### 4.1. Drugs and Reagents

(±)-α-tocopherol; methyl viologen dichloride hydrate; DMEM:F12 medium; penicillin–streptomycin solution hybri-max (P/S); fetal bovine serum (FBS); L-glutamine (L-Glut); non-essential amino acid solution (NEAA); trypan blue; AM281; WIN55,212-2; mesylate salt; 2-mercaptobenzothiazole; and 1,5-diaminophtalene were purchased from Sigma-Aldrich (Saint Louis, MO, USA). Methanol and acetonitrile were purchased from Panreac (Barcelona, Spain) and cesium chloride was purchased from HoneyWell (Charlotte, NC, USA).

### 4.2. Plant Material Cultivation and Extraction

#### 4.2.1. Plant Cultivation

Propagation from axillary or terminal buds was selected as the method for the tissue culture or in vitro propagation of different chemotypes of *Cannabis sativa* L. specimens. In order to ensure genetic and chemical stability during in vitro propagation, direct organogenesis was selected as the best micropropagation technique. This was because of the regeneration from existing meristems and the formation of new shoots without an intervening callus phase. Different combinations of modified Murashige and Skoog medium (MS), with and without plant growth regulators (PGRs), were selected and used to propagate different chemotypes of *Cannabis sativa* L. specimens. Shoots that were regenerated in vitro were rooted on modified Murashige and Skoog medium, which was supplemented with 1 mg of l-indole-3-butyric acid. All the cultures were grown under controlled conditions, at 25 °C ± 1 °C. The photoperiod consisted of 16 h of light and 8 h of dark. Light was provided by white fluorescent tubes of 18 W that provided 60 ± 5 µmol m^−2^ s^−1^ light intensity. Finally, the culture medium was replaced every 4–5 weeks. All plants employed in this research study were grown under a license for the cultivation of *C. sativa* for research purposes, issued by the Spanish Ministry of Health, Social Services, and Equality via the Spanish Agency of Medicines and Health Products (Agencia Española de Medicamentos y Productos Sanitarios or AEMPS) to ALEOVITRO Ltd (AleoVitro, Zamudio, Spain).

#### 4.2.2. Extraction and Reconstitution of Plant Extracts

The plants were allowed to flower under the environmental conditions described, and then they were collected and dried at 10–15% humidity. Next, the inflorescences were subjected to Soxhlet extraction with ethanol. The solvent was eliminated in vacuo to leave the extracts, with yields between 32 and 21%. Some of the plant extracts were reconstituted for HPLC–mass spectrometry analysis (described below), and other aliquots were reconstituted in absolute methanol, at a final concentration of 100 mg/mL of plant extract.

### 4.3. HPLC–Mass Spectrometry Analysis

The extracts were reconstituted with absolute methanol to a concentration of 1 mg/mL and analyzed by HPLC–PDA in a Shimadzu unit with an LC-20AD pump and a CTO-10AS VP column oven, coupled to an SPD-M20A Diode Array Detector. Separation was obtained using an ACE 3 C18 column (150 mm × 4.6 mm, 3 µm particle size) with an ACE3 C18 analytical pre-column. Compounds were eluted with methanol 0.1% acetic acid (LC–MS grade) (MeOH): MiliQ water 0.1% acetic acid, with a gradient of 80:100% over 15 min, 100% MeOH over 5 min, and 100:80% for 10 min, with a flow of 0.5 mL/min. The results were analyzed at a UV wavelength of 210 nm. The stock solutions of the ethanolic extracts were injected at 1 mg/mL, carrying out a 10 µL injection through an automatic injector (SIL-20A XR, Shimadzu, Kyoto, Japan). All extracts were dissolved in 100% MeOH for injection. The identification of the products was carried out through a comparison between the retention time and UV spectrum of standards of the pure product, previously injected. The percentage expressed in the table of the products is the relative percentage of each product of the whole extract, excluding the peaks of the sample with a percentage lower than 0.011%.

### 4.4. Cell Culture and Treatments

The human neuron cell line, SHSY-5Y, was cultivated in 12-well plates in complete medium (DMEM:F12 medium; 15% fetal bovine serum (FBS); 1% L-glutamine (L-Glut); 1% of non-essential amino acid solution (NEAA); and 1% penicillin–streptomycin solution hybri-max (P/S)) for 24 h, at 37 °C, 5% CO_2_, and constant humidity. Cells were habituated to low-serum conditions (DMEM:F12; 0.2% charcoal-treated FBS; 1% L-Glut; 1% NEAA; and 1% P/S) for 12 h before treatments began. After adaptation, different concentrations of cannabinoid extract were tested (1 μg/mL; 10 μg/mL; 30 μg/mL; and 100 μg/mL). In addition, a pre-treatment of 3 h, with or without α-tocopherol (1 μM) prepared in low-serum medium, was performed before treatment with or without cannabinoid extracts (3.5 μg/mL of plant extract concentration) in low-serum conditions, for 24 h. Moreover, cells were treated with synthetic cannabinoids WIN55,212-2 (3.5 μg/mL) or AM281 (3.5 μg/mL), with or without the α-tocopherol pre-treatment (1 μM). Cannabinoid extracts were diluted in absolute ethanol, while synthetic cannabinoids and α-tocopherol were diluted in DMSO. All tested situations had the same concentration of DMSO and absolute ethanol, to avoid changes due to vehicle solutions. After 24 h of cannabinoid treatment (either synthetic or plant extract), the viability assays were performed. Samples with only α-tocopherol pre-treatment were incubated with fresh low-serum medium, in the same manner as treated ones.

### 4.5. Viability Assay

To analyze the cellular viability at every condition and time point, trypan blue viability assays were performed. Cells were detached from the culture well using a mechanical method. The cell suspension was diluted 1:1 with 0.4% trypan blue solution, and cells were counted using a Neubauer chamber in an inverted microscope: Olympus CKX41 (Olympus Corporation, Tokyo, Japan). Dead cells (with a compromised membrane) were stained dark blue. The percentage of dead cells with respect to total cells was calculated.

Viability data handling and analysis was carried out using Excel and GraphPad software (version 9.2). Briefly, cell viability data were presented as a percentage of cell growth. The identification of outliers was carried out by applying the following formulas:

$$Y_{1}=\bar{X}-DF*SD\;\;\;\;\;\;\;\;\;Y_{2}=\bar{X}+DF*SD$$

*SD*—standard deviation; *DF*—deviation factor.

Points were identified as outliers and excluded if *Y*_1_ was higher than the point analyzed, or *Y*_2_ was lower than the point examined. We used a deviation factor of 1.25 in our analysis. Data were expressed as means of independent data points ± S.E.M. The results were analyzed using one-way, two-tailed, two-way ANOVA, with Tukey’s post-hoc. Statistical differences were indicated with *p*-values ≤ 0.05.

### 4.6. Cytochrome c Oxidase Activity Assay

The human neuron cell line, SHSY-5Y, was cultivated in 75 cm^2^ well plates, in complete medium (DMEM:F12 medium; 15% fetal bovine serum (FBS); 1% L-glutamine (L-Glut); 1% of non-essential amino acid solution (NEAA); and 1% penicillin–streptomycin solution hybri-max (P/S)), until they reached confluency. Cells were detached by a mechanical method to avoid enzymatic treatments (medium was changed to PBS at 4 °C and flasks were tapped laterally). Once cells were detached, they were pelleted and stored at −80 °C until use. The cell pellet was resuspended in phosphate buffer, pH 7.4, and passed through a 30Gx1/2″ syringe (0.3 mm × 12 mm), and then sonicated using a bath sonicator three times, for 10 s, to obtain the cell homogenate, and adjusted to 0.2 mg/mL solution in phosphate buffer (0.2 M, pH 7.4). The cell homogenate was added to the reaction solution (DAB 0.5 mg/mL and cytochrome C 0.01%) in a 96-well plate, in the presence or absence of *Cannabis sativa* plant extracts (final concentrations of the whole extract: 1 μg/mL, 10 μg/mL, and 100 μg/mL; concentrations of CBD and THC are indicated in Table 2). The reaction was measured at 450 nm, every 5 min, for 16 h. The linear part of the reaction curves was analyzed and slopes were obtained for each concentration.

### 4.7. MALDI-MS

Cells were detached by a mechanical method to avoid enzymatical treatments (medium was changed to PBS at 4 °C and flasks were tapped laterally). The cell suspension was passed through a 30Gx1/2″ syringe (0.3 mm × 12 mm) to produce a cell homogenate and then pelleted. A matrix-saturated solution was prepared by diluting 2-mercaptobenzothiazole (MBT) in a solution 3:5 of CsCl aqueous 200 mM:MeOH (final concentration of CsCl 75 mM). For positive-ion mode and 1,5-diaminopthtalene (DAN), a 1:1 solution of acetonitrile: distilled water was used. For each ionization mode, 2 μL of cell homogenate was added to 20 μL of matrix solution, 1 μL of each of this solution was spotted onto a sample plate, and each sample was spotted in triplicate.

The mass spectrometer used was an LTQ-Orbitrap XL (Thermo Fisher Scientific, Waltham, MA, USA), equipped with a MALDI source with a N_2_ laser (60 Hz, 100 μJ/pulse maximum power output). The laser spot was an ellipsoid of approximately 50–60 μm × 140–160 μm. Two microscans of 10 shots/spot were used, with a laser power output of 20 μJ for MS+ and 30 μJ for MS𲈒. Data loading included spectra normalization by total ion current (TIC), spectra alignment, and peak picking, filtering all the *m*/*z* with intensity <0.5% of the strongest peak in the spectrum.

MALDI spectra were aligned using MATLAB (Matworks, Natick, MA, USA), and lipids’ assignment was performed using a homemade database and the Lipid Maps LMSD database. For MALDI data analysis, MS+ and MS− data were normalized separately and then analyzed together. Matrix peaks and isotopic distribution were removed, and the remaining peaks were normalized against their total ion current (TIC). MS+ and MS− data were standardized using the z-score method, using the following formula.

$$Z=\frac{x-\mu }{\sigma }$$

*x*—observed value; *μ*—mean of the sample; *σ*—standard deviation of the sample.

## 5. Conclusions

In conclusion, cannabinoid extracts from high-CBD strains exhibit differential antitumor effects in human neuroblastoma cells by interfering with mitochondrial respiration and increasing ROS production, lipid peroxidation, and cell apoptosis in such a way that appears to correlate with THC content. However, the contributions of other compounds cannot be excluded.

This action of the *Cannabis sativa* plant extracts used in our study was not only mediated by the cannabinoid receptor’s activity, but also by its effect on the mETC complexes, among others, which promote oxidative stress. Moreover, the use of plant extracts has demonstrated higher antitumoral effects than cannabinoids by themselves [37]. However, the content of the antioxidants used in these extracts was not considered. This may also have contributed to the reduction in their effects, according to our data from the α-tocopherol pre-treatment.

This well-known antioxidant, which can protect against oxidative stress conditions through its scavenger action, is commonly administered to ameliorate the toxic effects of chemotherapy [49], which, according to our results, would be counterproductive in antitumor *Cannabis* treatments, as it would significantly reduce their efficacy.

## Figures and Tables

**Table 1:** Percentage of relative area of CBD, CBDA, THC, and THCA in different *Cannabis sativa* plant extracts.

Sample	CBD (%)	CBDA (%)	THC (%)	THCA (%)
P0-3	64.16	1.93	6.22	1.01
D2-2	60.16	1.05	5.65	0.21
D2-7	61.21	1.49	8.95	0.42

**Table 2:** Amount of CBD and THC content in every plant extract tested (P0-3, D2-2, and D2-7), at three different concentrations of whole extract (1 μg/mL, 10 μg/mL, and 100 μg/mL).

	CBD 1 μg/mL (μg/mL)	THC 1 μg/mL (μg/mL)	CBD 10 μg/mL (μg/mL)	THC—10 μg/mL (μg/mL)	CBD—100 μg/mL (μg/mL)	THC—100 μg/mL (μg/mL)
P0-3	0.6416	0.0622	6.416	0.622	64.16	6.22
D2-2	0.6016	0.0565	6.016	0.565	60.16	5.65
D2-7	0.6121	0.0895	6.121	0.895	61.21	8.95

## Data Availability

The data supporting the findings of this study are available from the corresponding author, Gabriel Barreda-Gómez, upon reasonable request.
